# The health-related quality of life of Brazilians with epidermolysis bullosa

**DOI:** 10.1186/s13023-025-03753-w

**Published:** 2025-07-04

**Authors:** Camille Cristine Gomes Togo, Ana Paula Caio Zidório, Vivian Siqueira Santos Gonçalves, Eliane Said Dutra

**Affiliations:** 1 Graduate Program in Human Nutrition, Faculty of Health Sciences, University of Brasilia, Campus Universitario Darcy Ribeiro, Brasilia, Federal District Brazil; 2 Clinical Nutrition Unit, University Hospital of Brasilia, Brasilia, Federal District Brazil; 3 Graduate Program in Public Health, Faculty of Health Sciences, University of Brasilia, Campus Universitario Darcy Ribeiro, Brasilia, Federal District Brazil

**Keywords:** Epidermolysis bullosa, Health-related quality of life, Children's dermatology life quality index, Quality of life evaluation in epidermolysis bullosa

## Abstract

**Background:**

Epidermolysis bullosa (EB) presents various complications that can affect an individual’s health-related quality of life (HRQoL) and nutritional status. This cross-sectional study evaluates HRQoL and its association with the socio-demographic, nutritional, and clinical profiles of Brazilians with EB.

**Results:**

The sample consisted of 129 individuals (median age: 15 years), approximately 57% female, mostly composed of the EB simplex clinical type, followed by the dystrophic EB type (DEB). The mean score on the Children’s Dermatology Life Quality Index questionnaire was 11.01 [standard deviation (SD) = 7.31], with a moderate impact on HRQoL. The median and interquartile range (IQR) of the score obtained in the Quality of Life Evaluation in Epidermolysis Bullosa– Brazilian Portuguese questionnaire was 13 (IQR: 8–19), which also indicated a moderate impact on HRQoL. In the subgroup aged ≥ 17 years, schooling positively influenced HRQoL, and decreased HRQoL was directly associated with underweight nutritional status. Family income promoted HRQoL in both subgroups.

**Conclusions:**

HRQoL assessment showed that the DEB type was associated with the most severe effect. Additionally, the large impact, in < 17 years, was associated with family income, but did not present an association with nutritional status. While the severe impact on HRQoL, in ≥ 17 years, presented an association with nutritional status, family income, and education. Future research is important, to understand this population’s needs, provide adequate care, and promote the best HRQoL.

**Supplementary Information:**

The online version contains supplementary material available at 10.1186/s13023-025-03753-w.

## Background

Epidermolysis bullosa (EB) is a rare heterogeneous genetic disorder, characterised by skin fragility, with the formation of blisters, erosions, and wounds on the skin and mucous membranes as a result of minor mechanical trauma [1, 2]. EB is classified into four main groups, according to the level of blister formation: EB simplex (EBS), junctional EB (JEB), dystrophic EB (DEB), and Kindler EB (KEB) [1, 3]. Data on the incidence and prevalence of EB are scarce, with 20 cases/million live births and 11 cases/million inhabitants in the United States [4]; 41.3 cases/million live births and 22.4 cases/million inhabitants in the Netherlands [5]; 45 cases/million live births and an estimated prevalence of 54 cases/million inhabitants in Germany [6]. Although there is no official epidemiological data in Brazil [7], the DEBRA Brazil (an association of people with EB in Brazil), has registered more than 800 individuals in its National Registry since 2014 [8].

The disease has diverse manifestations, affecting the skin, oral cavity, external eye, gastrointestinal tract, genitourinary tract, musculoskeletal system, upper airway, bone marrow, and heart [9]. These complications vary according to the classification of the clinical type of EB, compromising both health-related quality of life (HRQoL) and nutritional status [10].

HRQoL is defined as the individual’s perception of their life status given their disease, its consequences, and treatments [11]. The criteria for HRQoL evaluation include physical, psychological, and social health [12, 13]. These aspects are relevant for assessing disease severity, improvement through interventions, and allocation of resources [14].

Nutritional status is partially related to the form of EB, being generally more compromised in severe forms of recessive dystrophic EB subtype (RDEB) and JEB [15, 16]. As malnutrition leads to failure to thrive, delayed puberty, and anaemia, in addition to clinical and biological events that affect wound healing and increase skin breakdown in individuals with EB, continuous monitoring of nutritional status is a disease control strategy [17–19].

Epidemiological and clinical information may promote understanding of this population in Brazil and in other countries, mainly those in development, beyond strategies to address the condition. Considering the scarcity of information on HRQoL and socio-demographic, nutritional, and clinical profiles in individuals with EB in Brazil, this study aimed to evaluate HRQoL and its associations with socio-demographic, nutritional, and clinical profiles of Brazilians with EB.

## Methods

This study was conducted following the standards of Strengthening the Reporting of Observational Studies in Epidemiology (STROBE) [20].

### Study design and setting

The study design was observational and cross-sectional. The research was developed using the SurveyMonkey^®^ platform to deploy questionnaires on socio-demographic, nutritional, clinical, and HRQoL status.

### Data source

According to records available on the DEBRA Brazil website, nine regional EB associations across the country were contacted to raise awareness of the participation of individuals with EB in the research.

The data collection period was from October 2018 to October 2020. The associations of individuals with EB from the states of Brazil and the general population were informed by disseminating messages about the research through the media and by e-mailing individuals in the National Registry of DEBRA Brazil. The questionnaires were made available through social networks and the DEBRA Brazil website, with periodic reinforcement.

### Participants

Residents of Brazil, of both sexes, with EB regardless of the clinical type and physical condition, were included, with help provided by parents, caregivers, or legal guardians to complete the questionnaires. Children < 4 years old were excluded from the HRQoL assessment because of the lack of a suitable questionnaire at the time of data collection.

### Instruments and variables

The outcome variable was HRQoL, whereas the possible factors associated with HRQoL were socio-demographic, clinical, and nutritional.

### Socio-demographic, nutritional, and clinical profiles

A structured questionnaire was designed to obtain information on the date of birth, sex, family income, education level, region of the country, clinical type of EB, diagnosis, daily care for EB, use of nutritional supplements, and outpatient follow-up. The anthropometric assessment consisted of a self-report of the most recent weight and height, which is typically known by this population, given their routine health monitoring [21–23]. The classifications of underweight, eutrophy and overweight for nutritional status were according to the World Health Organization (WHO) references. For children and adolescents, the body mass index (BMI)-for-age curves from the WHO 2006 and 2007 were used [24, 25], and for adults, the WHO BMI classification (1995) was used [26].

### HRQoL

#### Children’s dermatology life quality index (CDLQI)

No specific questionnaire for children with EB was available during the study period; hence, the CDLQI was used to assess HRQoL in participants aged 4–16 years. This questionnaire was validated for Brazilian Portuguese, composed of ten questions about symptoms, leisure, school or holiday period, personal relationships, sleep, and treatment in the last week. It is highly internally consistent, test- and retest-reliable, responsive to change and significantly correlated with other objective and subjective measures [27]. Each question is rated from 0 to 3, with a maximum total score of 30 points. The higher the score, the more affected the individual’s HRQoL [28]. Scores are interpreted as follows: 0–1 (no effect on HRQoL), 2–6 (small effect), 7–12 (moderate effect), 13–18 (very large effect), and 19–30 (extremely large effect) [29].

### Quality of life evaluation in epidermolysis bullosa– Brazilian Portuguese (QoLEB– BP)

To assess HRQoL in individuals ≥ 17 years, the QoLEB-BP was used, which is a translation of The Quality of Life Evaluation in EB (QOLEB) [30] that has been culturally adapted and validated for Brazilian individuals [31]. This instrument is composed of seventeen questions that assess the functional and emotional aspects of individuals with EB. It was the first tool to measure the quality of life specific to EB and is considered a valid and reliable instrument for quantifying it in patients with different subtypes of EB. Furthermore, the QOLEB may be used as a sensitive tool to monitor the quality of life and to highlight dimensions as potential targets for interventions and research [30]. Each question is rated from 0 to 3, with a maximum total score of 51. The total score is interpreted as follows: 0–4 (very mild impact on HRQoL), 5–9 (mild impact), 10–19 (moderate impact), 20–34 (severe impact), and 35–51 (very severe impact) [32].

### Data analysis

The database was analysed using Stata^®^ statistical software, version 16.1 [33]. The analysis was conducted on the complete sample and stratified into two subgroups (< 17 years and ≥ 17 years).

The Kolmogorov-Smirnov normality test was performed on the quantitative variables, which were not normally distributed and for this reason non-parametric tests have been used. The variables are presented as median and interquartile range (IQR), while categorical variables are presented as prevalence and confidence intervals (CI 95%).

Poisson regression with robust variance was used to estimate the prevalence ratio. Crude analyses were performed for each variable; if the p-value was < 0.20, adjusted analyses were performed according to age, sex, and clinical type of EB. Statistical significance was defined as *p* < 0.05.

### Ethical considerations

Before participants completed the questionnaires, a Free and Informed Consent Form was presented so that participants only answered the questions after they had read and accepted the terms of consent. This study was approved by the Human Research Ethics Committee of the Health Sciences Faculty of the University of Brasília (protocol no. 2.870.738).

## Results

**Fig. 1:**
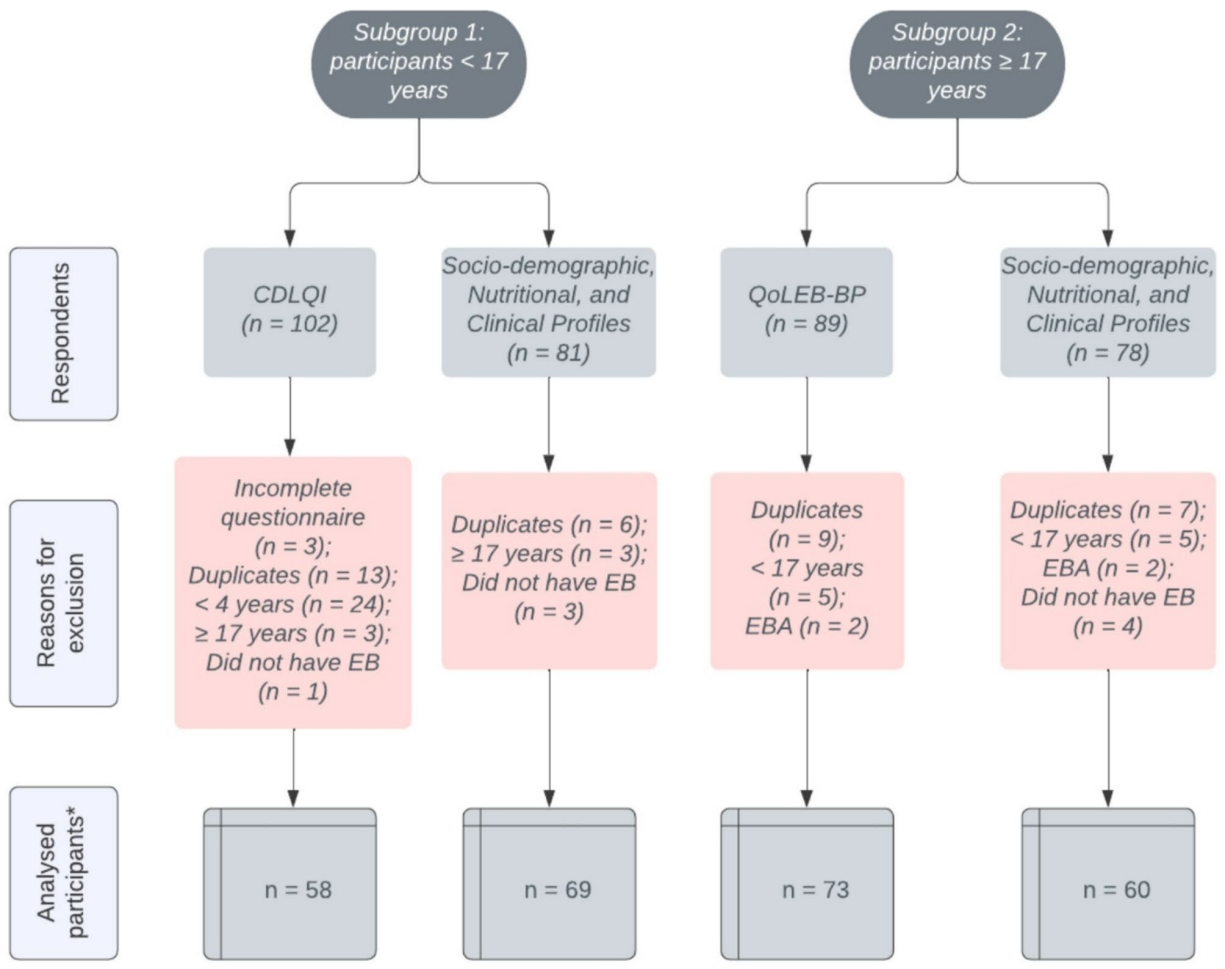
Flowchart of the sample of participants. *The number of analysed participants differs between the questionnaires, as some individuals answered both questionnaires (health-related quality of life and socio-demographic, nutritional and clinical profiles), but others answered only one of the research questionnaires. CDLQI: Children’s Dermatology Life Quality Index; QoLEB-BP: Quality of Life Evaluation in Epidermolysis Bullosa– Brazilian Portuguese; EBA: epidermolysis bullosa acquisita

A total of 191 individuals with EB responded to the initial invitation, but not all were included in all analyses (Fig. 1).

The total sample encompassed 129 individuals, approximately 57% female, with a median age of 15 years. Most participants had EBS, followed by DEB; one person was diagnosed with KEB. More than half of the sample (63.57%) received outpatient follow-up, approximately 44% were eutrophic, and almost all (96.12%) received special daily care. In subgroup 1 (< 17 years), 100% had daily care consisting of skin hydration, dressing change, wearing comfortable clothes and shoes, and hygiene, whereas, in subgroup 2 (≥ 17 years), approximately 91.7% had the same care. As to managing blisters, a small number of participants described this daily care, in both subgroups. Approximately half of the participants received their diagnosis exclusively based on clinical criteria (Table 1).

**Table 1:** Socio-demographic, nutritional and clinical profiles of Brazilians with epidermolysis bullosa, 2018–2020

Variables	Total sample (*n* = 129) (median and interquartile or % and CI 95%)	< 17 years (*n* = 69) (median and interquartile or % and CI 95%)	≥ 17 years (*n* = 60) (median and interquartile or % and CI 95%)
Age (years)	15 (5; 27)	6 (2; 10)	28 (23; 38)
Female sex	56.59 (47.84; 64.94)	47.83 (36.17; 59.72)	66.67 (53.62; 77.58)
Family income ($)	494.36 (257.06; 988.71)	413.28 (206.64; 692.10)	593.23 (316.39; 988.71)
Schooling*			
Early childhood education/Did not study	29.69 (22.36; 38.24)	49.28 (37.53; 61.10)	6.78 (2.51; 17.02)
Elementary and Secondary education	50 (41.34; 58.66)	50.72 (38.90; 62.47)	49.15 (36.47; 61.95)
University education	-	-	44.07 (31.79; 57.11)
**Brazil region**			
North	6.2 (3.11; 11.98)	5.80 (2.15; 14.68)	6.67 (2.47; 16.75)
North East	10.85 (6.50; 17.57)	11.59 (5.83; 21.74)	10 (4.49; 20.81)
Midwest	11.63 (7.10; 18.47)	14.49 (7.89; 25.11)	8.33 (3.45; 18.80)
South	18.6 (12.75; 26.34)	20.29 (12.29; 31.63)	16.67 (9.09; 28.57)
Southeast	52.71 (44.02; 61.24)	47.83 (36.17; 59.72)	58.33 (45.33; 70.28)
**EB diagnosis**			
Exclusively clinical	51.16 (42.51; 59.75)	49.28 (37.53; 61.10)	53.33 (40.51; 65.73)
Clinical + Immunofluorescence mapping	19.38 (13.40; 27.19)	21.74 (13.43; 33.21)	16.67 (9.09; 28.57)
Clinical + Electron microscopy	6.2 (3.11; 11.98)	5.80 (2.15; 14.68)	6.67 (2.47; 16.75)
Clinical + Molecular genetic test	5.43 (2.59; 11.02)	8.70 (3.90; 18.26)	1.67 (0.22; 11.31)
Clinical + Biopsy**	17.83 (12.10; 25.49)	14.49 (7.89; 25.11)	21.67 (12.87; 34.12)
**Outpatient follow-up and care**			
Daily special care	96.12 (90.96; 98.39)	100	91.67 (81.20; 96.55)
Outpatient follow-up	63.57 (54.85; 71.48)	75.36 (63.66; 84.23)	50 (37.36; 62.64)
Nutritional supplement	45.74 (37.27; 54.46)	53.62 (41.67; 65.18)	36.67 (25.30; 49.74)
**Nutritional status**			
Underweight	28.85 (20.88; 38.38)	32.65 (20.81; 47.22)	25.45 (15.51; 38.84)
Eutrophy	44.23 (34.90; 53.99)	51.02 (36.96; 64.92)	38.18 (26.15; 51.86)
Overweight	26.92 (19.20; 36.35)	16.33 (8.23; 29.80)	36.36 (24.57; 50.06)

Data are presented as median and interquartile range (p25 and p75)

*Education levels can be complete or incomplete

** Individuals who marked this item did not know which method for analysis was used later, but there is an extra method used after the biopsy

### CDLQI and QoLEB-BP scores

For subgroup 1, the mean CDLQI score was 11.01 [standard deviation (SD) = 7.31, range: 0–25]. The items that contributed most to the final CDLQI score were related to symptoms/sensations and leisure (Additional file 1 - Appendix A). Of the participants, 93.1% reported itching, pain, or sensitivity of the skin; 67.2% felt embarrassed, upset, or sad; 63.8% needed to reduce or avoid swimming and playing sports; 46.5% had their friendships affected; 81% needed to wear special or different clothes/shoes; 67.2% had their outings, games, or hobbies disrupted; 50% had problems with people bullying, asking questions, and avoiding them; and 58.6% had sleep problems.

Individuals of subgroup 2 had a median QoLEB-BP score of 13 (IQR: 8–19, range: 0–40). The items that contributed most to the QoLEB-BP scores on the functional scale were financial impact, physical pain, and difficulty in practising sports. On the emotional scale, feelings of shame, anxiety, frustration, depression, and discomfort by being observed by strangers prevailed. Overall, participants in this subgroup were more affected functionally than emotionally (Additional file 2 - Appendix B).

Of the participants, 46.6% reported reduced ability to move around the house, 94.5% felt physical pain, 87.7% were affected in their practice of sports, 75.3% felt frustrated, 28.8% reported reduced ability to shower and write, 45.2% reported reduced ability to eat, 47.9% reported reduced ability to go shopping, 61.6% had diminished ability to move outside the home, 35.6% were affected in their relationship with family members, 65.7% felt ashamed, 24.6% needed to modify their house, 39.7% were affected in their relationship with friends, 78.1% felt anxious or worried, 69.9% were financially affected, 58.9% felt depressed, and 68.5% felt uncomfortable being observed or teased by strangers.

### HRQoL

Table 2 presents HRQoL by EB clinical type, and by age category. In subgroup 1 (*n* = 58), the DEB type prevailed (36.2%). In the evaluation of HRQoL, 37.93% had a very large or extremely large effect, with the most frequent being DEB type (45.45%). In subgroup 2 (*n* = 73), the EBS type was prevalent (43.83%). In the HRQoL assessment, 24.65% of the sample presented a severe or very severe impact due to EB, with the DEB type also being more frequent (38.88%).

**Table 2:** Assessment of health-related quality of life according to clinical type in Brazilians with epidermolysis bullosa, 2018–2020

< 17 years (CDLQI)						
EB clinical type\Impact on HRQoL (score)	**EBS** **(** ***n*** ** = 17)** **n (%)**	**DEB** **(** ***n*** ** = 21)** **n (%)**	**JEB** **(** ***n*** ** = 2)** **n (%)**	**Do not know** **(** ***n*** ** = 1)** **n (%)**	**NI** **(** ***n*** ** = 17)** **n (%)**	**Total** **(** ***n*** ** = 58)** **n (%)**
No effect (0–1)	0 (0)	1 (1.72)	0 (0)	0 (0)	6 (10.34)	7 (12.1)
Small effect (2–6)	3 (5.17)	5 (8.62)	0 (0)	0 (0)	4 (6.89)	12 (20.7)
Moderate effect (7–12)	9 (15.51)	5 (8.62)	0 (0)	0 (0)	3 (5.17)	17 (29.3)
Very large effect (13–18)	0 (0)	6 (10.34)	1 (1.72)	0 (0)	4 (6.89)	11 (18.9)
Extremely large effect (19–30)	5 (8.62)	4 (6.89)	1 (1.72)	1 (1.72)	0 (0)	11 (18.9)
**≥ 17 years (QoLEB-BP)**						
**EB clinical type/** **Impact on** **HRQoL (score)**	**EBS** **(** ***n*** **= 32)** **n (%)**	**DEB** **(** ***n*** **= 20)** **n (%)**	**KEB** **(** ***n*** **= 1)** **n (%)**	**Do not know** **(** ***n*** **= 4)** **n (%)**	**NI** **(** ***n*** **= 16)** **n (%)**	**Total** **(** ***n*** **= 73)** **n (%)**
Very mild (0–4)	6 (8.21)	0 (0)	0 (0)	0 (0)	2 (2.73)	8 (11)
Mild (5–9)	8 (10.95)	4 (5.46)	1 (1.36)	0 (0)	3 (4.10)	16 (21.9)
Moderate (10–19)	15 (20.54)	9 (12.3)	0 (0)	2 (2.73)	5 (6.84)	31 (42.4)
Severe (20–34)	3 (4.10)	6 (8.19)	0 (0)	2 (2.73)	5 (6.84)	16 (22)
Very severe (35–51)	0 (0)	1 (1.36)	0 (0)	0 (0)	1 (1.36)	2 (2.7)

EB: epidermolysis bullosa; HRQoL: health-related quality of life; CDLQI: Children’s Dermatology Life Quality Index; QoLEB-BP: Quality of Life in Epidermolysis Bullosa-Brazilian Portuguese; EBS: epidermolysis bullosa simplex; DEB: dystrophic epidermolysis bullosa; JEB: junctional epidermolysis bullosa; KEB: Kindler epidermolysis bullosa; NI: not informed

### Associations between HRQoL and nutritional, socio-demographic variables, and outpatient follow-up

Table 3 presents crude and adjusted prevalence ratio (PR) for the association between nutritional, socio-demographic variables, outpatient follow-up and HRQoL. In the adjusted analysis of subgroup 1, a large impact on HRQoL was negatively associated with the highest range of family income [PR: 0.17; CI 95%: 0.05; 0.51]. No association was found between nutritional status and outpatient follow-ups. Considering the low median age of this subgroup, schooling was not analysed.

**Table 3:** Prevalence ratios for the association between nutritional, socio-demographic variables, outpatient follow-up and health-related quality of life in Brazilians with epidermolysis bullosa, 2018–2020

Large impact* on HRQoL					Severe impact** on HRQoL				
	< 17 years				≥ 17 years				
	Crude		Adjusted****		Crude			Adjusted****	
	PR*** **(CI 95%)**	**P-value**	PR*** **(CI 95%)**	**P-value**		PR*** **(CI 95%)**	**P-value**	PR*** **(CI 95%)**	**P-value**
**Nutritional status**									
Eutrophy	Ref.					Ref.		Ref.	
Underweight	0.96 (0.33; 2.72)	0.941	*****	*****		2.18 (0.71; 6.68)	0.170	3.06 (1.08; 8.67)	0.035
Overweight	0.5 (0.07; 3.49)	0.485	*****	*****		0.52 (0.10; 2.59)	0.429	*****	*****
**Schooling**									
Early childhood education / Did not study						Ref.		Ref.	
Elementary and secondary education						0.22 (0.06; 0.75)	0.016	0.11 (0.02; 0.66)	0.016
University education						0.34 (0.11; 1.01)	0.053	0.19 (0.02; 1.61)	0.129
**Family income ($)** ^**a**^					**Family income ($)** ^**a**^				
< 206.64	Ref.				< 316.39	Ref.		Ref.	
206.64 ≤ 692.10	0.78 (0.41; 1.49)	0.467	*****	*****	316.39 ≤ 988.71	0.28 (0.08; 0.95)	0.041	0.13 (0.03; 0.55)	0.006
> 692.10	0.23 (0.05; 0.91)	0.036	0.17(0.05; 0.51)	0.002	> 988.71	0.28 (0.08; 0.95)	0.041	0.17 (0.05; 0.57)	0.004
**Outpatient follow-up**									
No	Ref.					Ref.		Ref.	
Yes	0.95 (0.43; 2.07)	0.904	*****	*****		2.22 (0.74; 6.61)	0.152	1.98 (0.28; 13.65)	0.487

*Very large effect + Extremely large effect

**Severe impact + Very severe impact

***Prevalence ratio

****Adjusted by sex, age and EB clinical type

*****P-value ≥ 0,20 in crude analysis

^a^ The income ranges were different in the subgroups by age

In subgroup 2, severe impact on HRQoL was directly associated with underweight (PR: 3.06; CI 95%: 1.08; 8.67), with no association with overweight. A negative association of severe impact on HRQoL was found with elementary and secondary education (PR: 0.11; CI 95%: 0.02; 0.66), with no association with university education. A negative association of severe impact on HRQoL was found with middle (PR: 0.13; CI 95%: 0.03; 0.55) and higher (PR: 0.17; CI 95%: 0.05; 0.57) income brackets. No association was found with outpatient follow-up Additional file 3.docx.

## Discussion

This study is a pioneering assessment of HRQoL and its associated factors in individuals with EB in Brazil. Schooling had a positive influence on HRQoL in subgroup 2, and family income promoted better HRQoL in both subgroups. The prevalence of the clinical types of EB followed that observed in the Dutch registry of individuals with the disease; EBS was most prevalent, followed by DEB and JEB, with a lower prevalence of KEB [5].

Most participants resided in the Southeast Brazil region and the fewest in the North region. This reduced participation of the North region can be attributed to regional inequalities and lack of infrastructure, which affect telecommunication services in the region. Poor connection quality, limited coverage, and high cost of Internet access also contribute [34].

Although the Clinical Protocol and Therapeutic Guidelines for hereditary and acquisita EB state that diagnosis can be confirmed using biopsy or genetic tests, clinical diagnosis was predominant in the evaluated participants [35–37]. In Brazil, a small proportion of people with EB have access to their type of EB. Among the reasons are technical difficulties and the need for specialists to carry out the analysis. Another reason is the lack of reference health centres in Brazil, as well as the unavailability of tests in the public health system [38]. As a result, many Brazilians with the disease do not get an accurate and conclusive diagnosis of their type of EB, a limitation observed in our study. In contrast, other countries more frequently use confirmation by genetic tests or biopsy [39, 40]. Electron microscopy is less used; few laboratories worldwide perform this procedure to analyse EB [41]. However, it is necessary to understand that in the face of limited resources, clinical diagnosis has its value, as demonstrated by Yenamandra et al. (2017), who, seeing these difficulties in developing countries, developed a clinical diagnostic tool for the diagnosis and subtyping of EB [42].

Concerning the Brazilian scenario, in which these individuals are inserted, there is a National Policy for Comprehensive Care for People with Rare Diseases, with transversal coverage to the priority thematic networks of the Unified Health System (UHS). This policy aims to reduce mortality, and contribute to the reduction of morbidity and secondary manifestations, in addition to seeking to improve people’s quality of life, through actions of promotion, prevention, early detection, timely treatment, reduction of disability and care palliatives [43]. There is also the Brazilian guidelines for the care of patients with Epidermolysis Bullosa, aiming to establish the diagnostic and therapeutic criteria for epidermolysis bullosa [7]; beyond Guidelines for Comprehensive Care for People with Rare Diseases within the scope of the UHS, which addresses the healthcare network in this population, involving basic, home care, as well as specialized outpatient and hospital care [44].

About the country’s climate, according to the Brazilian Institute of Geography and Statistics, there are variations according to the region, which may be an equatorial, tropical or temperate climate. However, in general, Brazil has a hot climate, which facilitates the formation of blisters in individuals with EB, considering that the heat increases their appearance [45]. This is consistent with what was observed by Brun et al. (2017), in which a higher frequency of participants had pain during the summer season than in the winter season [46].

Chronic wounds are the focus of daily skin care, as they have a negative psychosocial impact on individuals with RDEB, as well as medical and financial burdens [47–51]. Additionally, Eng et al. (2021) [39] reported that higher QOLEB scores in individuals with RDEB were correlated with greater wound size. The International Consensus– Best Practice Guidelines– Skin and Wound Care in Epidermolysis Bullosa (2017) [52] brings the management of blisters as an essential part of skin and wound management, however, few participants have described this care in our study. This result shows the importance of emphasizing this daily care since the blisters are not self-limiting and may extend rapidly if left unchecked [52].

Nutritional status may vary depending on the clinical type of EB [10]. It is important to consider this aspect, as those most severely affected by the disease have a negative nutritional balance because of the limiting factors of nutritional consumption and hypermetabolism associated with open skin lesions, which leads to increased protein requirements and heat loss. Therefore, improvement of the HRQoL of these individuals requires wound management, infection control, pain relief, and improved individual nutritional status [53].

Retrosi et al. (2022) [54] described that HRQoL is highly affected by EB for several reasons, such as manifestation from birth, being a chronic disease, often being multisystemic, being associated with reduced life expectancy, and resulting in serious aesthetic damage. Additionally, EB is incurable and causes pain and weakness that requires daily attention and skin care [9, 45, 55].

The mean score obtained in subgroup 1 was higher in our study [11.01 (SD = 7.31)] than in Brun et al. (2017) [46] [8.1 (SD = 5.1)], which represents a moderate effect [29]. Additionally, the participants in our study were more affected by itching, pain, or skin sensitivity, as well as negative affect and the need to reduce or avoid the practice of sports, possibly due to the more severe types of EB included in our study, considering that Brun et al. (2017) only evaluated children with localised EBS (EBS-l) [46].

In subgroup 2, the mean QoLEB-BP score had a moderate effect on HRQoL [32]. The mean in our study was again higher than that in Brun et al. (2017) [46] [*n* = 30, only EBS-l type; QOLEB score: 6.6 (SD = 4.9 points)], possibly due to the most severe clinical types of EB included in our study. However, the mean in our study was lower than that in Eng et al. (2021) [39] [*n* = 39; QOLEB: 20 (SD = 9 points)], possibly because they only evaluated participants with RDEB, and in Yazdanshenas et al. (2020) [56] [*n* = 83; QOLEB: 43.7 (SD: 9.9 points)], possibly because most of their sample was composed of the DEB, as opposed to the EBS type in our study. Similar to Brun et al. (2017) [46] and Yazdanshenas et al. (2020) [56], a high percentage of individuals were affected in their practice of sports (87.7% vs. 87% vs. 100%), and felt physical pain (94.5% vs. 100% vs. 97.6%). In contrast, Brun et al. (2017) [46] found no difficulty with eating and bathing, whereas in our study, 28.8% had a reduced ability to shower and 45.2% had a diminished ability to feed.

Functional scale items contributed most to the final QoLEB-BP score and emotional aspects contributed least, a finding similar to another Brazilian study in two capitals [31]. High levels of resilience developed by more severely affected participants may explain this lower emotional effect [31, 56, 57]. Furthermore, children who accept the disease or distance themselves from it seem to feel better than those who engage in cognitive-palliative strategies or express emotional reactions [58].

The greatest impact on HRQoL was identified in the DEB type, similar to other studies, which found a greater impact in individuals with the RDEB subtype [30, 47, 57, 59, 60], as well as in a systematic review [61]. As in subgroup 2, HRQoL was primarily compromised via pain and difficulty in practising sports, emphasising the need to assess this aspect dynamically and in different populations [61].

An inverse association was identified between severe impact on HRQoL and schooling in subgroup 2. Additionally, in both subgroups, an inverse association was found between income and severe/large impact on HRQoL. This is possibly related to individuals with higher family incomes having better access to the necessary care and monitoring of the disease, given the high financial burden of EB. According to a systematic review by Tang et al. (2021) [62], the total annual medical costs are $84,534 in Ireland and $7,392 in Korea. The estimated annual costs for dressings in the United States range from $4,000 to $245,000. Individuals with RDEB and their caregivers have significant economic, humanistic, and clinical challenges.

Although studies have shown nutritional impairment in people with EB since childhood [63–65], unexpectedly, no association was found between the large impact on HRQoL and nutritional status in the subgroup aged < 17 years in our study. This may be a result of the difference in care between children and adults with EB. The child can receive better care, since they are dependent on an adult, often engaged in improving the child’s quality of life. However, adults with EB, who need to take care of themselves, may end up neglecting themselves as a result of other daily life activities. It would be interesting to conduct more in-depth studies to investigate the association between HRQoL and nutritional status in this population, to better understand this result.

The originality of this study to Brazil is a strength, as its inclusion of participants from all regions of the country, representation of all clinical types of EB, and analysis using robust statistical methods. However, its limitations include difficulty in contacting individuals with EB registered by existing associations; and the use of an online questionnaire, making it impossible to guarantee its completion. The online survey also prevented a more precise estimate of participants’ nutritional status, once it was made exclusively by the BMI estimative with the referred height and weight. This points to the need to carry out in-depth technical estimation of patients’ nutritional status. Finally, the cross-sectional design, which prevented causality from being established.

## Conclusions

Although there are rare disease care guidelines in the UHS and diagnostic and therapeutic criteria for EB (the Guidelines for Comprehensive Care for People with Rare Diseases in the UHS [44] and the Brazilian Guidelines for the Care of Patients with EB [7]), this study is the first to evaluate HRQoL and its associated factors, using the socio-demographic, clinical, and nutritional profiles of Brazilians with EB. HRQoL assessment showed that the DEB clinical type was the most severely affected. Also, the large impact on HRQoL was negatively associated with family income and did not present an association with nutritional status in < 17 years. While the severe impact on HRQoL presented a direct association with poor nutritional status and an inverse association with family income and schooling in ≥ 17 years. Given these findings, we note the importance of further research on the HRQoL of individuals with EB. There is also a need for improved care protocols, guidelines, and treatment of individuals with EB, considering the particularities of the Brazilian population, to promote better HRQoL. As to other clinical data of EB, such as the gravity score of the disease and its complications, and correlation with HRQoL, we have discussed in another article of our group, entitled: Health-related quality of life and clinical severity in people with epidermolysis bullosa– a proposal for assessing nutritional compromise by body mass index (Birmingham Epidermolysis Bullosa Severity score) [66].

## Electronic supplementary material

Below is the link to the electronic supplementary material.

Supplementary Material 1

Supplementary Material 2

## Data Availability

All data generated or analysed during this study are included in this published article [Appendix (A) Distribution of scores on each of the CDLQI items (*n* = 58); Appendix (B) Distribution of scores on each of the QoLEB-BP items (*n* = 73)].

## Abbreviations

BMI: Body mass index
CDLQI: Children’s Dermatology Life Quality Index
CI: Confidence interval
DEB: Dystrophic epidermolysis bullosa
EB: Epidermolysis bullosa
EBS: Epidermolysis bullosa simplex
EBS-l: Localised epidermolysis bullosa simplex
HRQoL: Health-related quality of life
IQR: Interquartile range
JEB: Junctional epidermolysis bullosa
KEB: Kindler epidermolysis bullosa
PR: Prevalence ratio
QOLEB: Quality of Life Evaluation in Epidermolysis Bullosa
QoLEB BP: Quality of Life Evaluation in Epidermolysis Bullosa– Brazilian Portuguese
RDEB: Recessive dystrophic epidermolysis bullosa
SD: Standard deviation
UHS: Unified Health System
WHO: World Health Organization
