# Supplementary material for: The health-related quality of life of Brazilians with epidermolysis bullosa
Source: Orphanet J Rare Dis. 2025 Jul 4;20:341. doi: 10.1186/s13023-025-03753-w (PMC12228413; doi:10.1186/s13023-025-03753-w)
Supplement: Supplementary file 1 — Supplementary Material 1 [file 13023_2025_3753_MOESM1_ESM.docx]

**Appendix A.** Distribution of scores on each of the CDLQI items (*n* = 58)

| CDLQI items | Scored 0 (*n*) | Scored 1 (*n*) | Scored 2 (*n*) | Scored 3 (*n*) |
| --- | --- | --- | --- | --- |
| 1 | 4 | 27 | 16 | 11 |
| 2 | 19 | 21 | 13 | 5 |
| 3 | 31 | 18 | 7 | 2 |
| 4 | 11 | 16 | 19 | 12 |
| 5 | 19 | 14 | 14 | 11 |
| 6 | 21 | 9 | 16 | 12 |
| 7a* | 13 | 11 | 3 | 8 |
| 7b* | 6 | 2 | 0 | 1 |
| 8 | 29 | 16 | 9 | 4 |
| 9 | 24 | 16 | 10 | 8 |
| 10 | 23 | 20 | 12 | 3 |

1. Over the last week, how **itchy**, **"scratchy"**, **sore** or **painful** has your skin been?; 2. Over the last week, how **embarrassed** or self-conscious, **upset** or **sad** have you been because of your skin?; 3. Over the last week, how much has your skin affected your **friendships**?; 4. Over the last week, how much have you changed or worn **different** or **special** **clothes/shoes** because of your skin?; 5. Over the last week, how much has your skin trouble affected **going out**, **playing**, or **doing hobbies**?; 6. Over the last week, how much have you avoided **swimming** or **other sports** because of your skin trouble?; 7a. **If school time**: Over the last week, how much did your skin problem affect your **school work**?; 7b. **If holiday time**: How much over the last week, has your skin problem interfered with your enjoyment of the **holiday**?; 8. Over the last week, how much trouble have you had because of your skin with other people **calling you names**, **teasing**, **bullying**, **asking questions** or **avoiding you**?; 9. Over the last week, how much has your **sleep** been affected by your skin problem?; 10. Over the last week, how much of a problem has the **treatment** for your skin been?

*This item was not applicable for 13 individuals, because they were not of school age and one individual did not respond.
