# Supplementary material for: The health-related quality of life of Brazilians with epidermolysis bullosa
Source: Orphanet J Rare Dis. 2025 Jul 4;20:341. doi: 10.1186/s13023-025-03753-w (PMC12228413; doi:10.1186/s13023-025-03753-w)
Supplement: Supplementary file 2 — Supplementary Material 2 [file 13023_2025_3753_MOESM2_ESM.docx]

**Appendix B.** Distribution of scores on each of the QoLEB-BP items (*n* = 73)

| QoLEB-BP items | Scored 0 (*n*) | Scored 1 (*n*) | Scored 2 (*n*) | Scored 3 (*n*) |
| --- | --- | --- | --- | --- |
| 1 | 39 | 29 | 2 | 3 |
| 2 | 52 | 8 | 2 | 11 |
| 3 | 4 | 48 | 13 | 8 |
| 4 | 52 | 11 | 8 | 2 |
| 5 | 40 | 24 | 9 | 0 |
| 6 | 38 | 26 | 6 | 3 |
| 7 | 9 | 19 | 30 | 15 |
| 8 | 18 | 38 | 11 | 6 |
| 9 | 28 | 34 | 8 | 3 |
| 10 | 47 | 23 | 3 | 0 |
| 11 | 25 | 35 | 12 | 1 |
| 12 | 55 | 12 | 2 | 4 |
| 13 | 44 | 25 | 4 | 0 |
| 14 | 16 | 33 | 19 | 5 |
| 15 | 22 | 28 | 17 | 6 |
| 16 | 30 | 29 | 12 | 2 |
| 17 | 23 | 29 | 19 | 2 |

1. Does your EB affect your ability to move around at home?; 2. Does your EB affect your ability to bathe or shower?; 3. Does your EB cause you physical pain?; 4. How does your EB affect your ability to write?; 5. Does your EB affect your ability to eat?; 6. Does your EB affect your ability to go shopping?; 7. How does EB affect your involvement in sports?; 8. How frustrated do you feel about your EB?; 9. Does your EB affect your ability to move around outside of your home?; 10. How does your EB affect your relationships with family members?; 11. How embarrassed do people make you feel about your EB?; 12. Have you needed to, or do you need to modify your home (installing ramps etc.) due to your EB?; 13. Does your EB affect your relationships with friends?; 14. How worried or anxious do you feel because of your EB?; 15. How are you or your family affected financially by your EB?; 16. How depressed do you feel because of your EB?; 17. How uncomfortable are you made to feel by others (e.g. teasing or staring) because of your EB?
